# Resin infiltration and fluoride varnish for caries control in children: A systematic review

**DOI:** 10.6026/973206300213979

**Published:** 2025-10-31

**Authors:** Kshitija Kamlakar Bansode, Nageshnath Baliram Waghmare, Aditya Vitthal Kulkarni, Rutuja Vikram Nalwade, Pratiksha Prakash Pawar, Anusha Jayan

**Affiliations:** 1 Department of Dentistry, Government Medical College and Hospital, Dharashiv, Maharashtra, India; 2 Department of Prosthodontics and Crown & Bridge, Government Dental College and Hospital, Jalgaon, Maharashtra, India; 3 Department of dentistry, Vasant Dada Patil Dental College and Hospital, Kavlapur, Sangli, Maharashtra, India; 4 Department of dentistry, Government Dental College and Hospital, Mumbai, Maharashtra, India

**Keywords:** Children, dental caries, resin infiltration, fluoride varnish

## Abstract

Dental caries progression in children remains a major public health concern despite widespread fluoride use. Therefore, it is of interest to compare the effectiveness of resin infiltration combined with fluoride varnish versus fluoride varnish alone in reducing caries progression among school children. The combination therapy showed greater efficacy in arresting and reducing carious lesion progression than fluoride varnish alone. Thus, resin infiltration with fluoride varnish offers superior protection against dental caries in children.

## Background:

Dental caries is one of the most common yet preventable diseases in children [1, 2, 3-4]. It remains a primary cause of oral pain and tooth loss [3, 4- 5]. Although the prevalence of carious lesions in children and adolescents has declined over the past 30 years [6, 7], it continues to be a significant oral health problem. In the primary dentition, the progression rate of enamel carious lesions is reported to be 2-3 times faster than in the permanent dentition [8, 9]. Moreover, caries progression in dentin occurs 3-6 times more rapidly than in enamel, irrespective of the dentition type [8, 9, 10-11]. Dental caries can be arrested or reversed in its early stages; however, it is not self-limiting and, without appropriate intervention, can progress to extensive tooth destruction [4]. Strong evidence supports the role of fluoride as the cornerstone of caries prevention, in conjunction with patient education and routine follow-up. Fluoride is the only compound officially recognized by the U.S. Food and Drug Administration (FDA) for dental caries prevention, though not all fluoride-containing products have FDA approval for this purpose. The primary sources of fluoride include fluoridated community water, fluoride-containing toothpaste and mouth rinses [12]. Fluoride varnishes, introduced in the late 1960s, were developed to enhance the effectiveness of topical fluoride by prolonging contact between the enamel surface and fluoride ions. For preschool children, professionally applied fluoride varnish remains the most effective and practical topical fluoride method [13, 14]. Resin infiltration represents a modern micro-invasive technique aimed at preventing and stabilizing non-cavitated carious lesions extending up to the first third of dentin (D1). Marketed as Icon® (DMG America, Englewood, NJ), it is designed to infiltrate and reinforce demineralized enamel without sacrificing healthy tissue [15, 16]. The technique relies on resin penetration into porous enamel via capillary action, thereby occluding micro-porosities that serve as diffusion pathways for acids and minerals. This creates a diffusion barrier within the lesion rather than merely on its surface [17, 18]. Despite promising results, few studies have directly compared the caries-preventive efficacy of resin infiltration versus fluoride varnish. Therefore, it is of interest to compare and evaluate the efficacy of resin infiltration with fluoride varnish versus fluoride varnish alone in reducing dental caries progression in the deciduous dentition among school children.

## Materials and Methods:

## Protocol registration:

The Preferred Reporting Items for Systematic Reviews and Meta-Analyses (PRISMA) [19] protocols were referred to conduct this review and the same was registered at National Institute for Health Research PROSPERO International Prospective Register for Systematic Reviews (CRD42021236904).

## Review question:

The population (P), intervention (I), comparison (C) and outcome (0) framework were used to generate the research question. Is Resin infiltration with Fluoride varnish more effective in reducing dental caries, when compared to Fluoride varnish alone in children?

## Search strategy:

Electronic databases like EBSCOhost, Cochrane Library and PubMed were searched. Methodological Medical Subject Heading (MeSH) words were constructed using the PICO-formatted question. For each database, these techniques were tweaked as needed. The Cochrane Highly Sensitive Search Technique (CHSSS) was used to locate randomized trials and the search strategy included controlled vocabulary and free text words (RCTs).

## Inclusion criteria:

The inclusion criteria for the present review were - Peer reviewed scientific journals from 2005 to 2020, full articles in English were included, randomized Controlled trial, studies which include Resin infiltration and Sodium Fluoride versus Sodium fluoride and any other group, school children of any age group, IACD Criteria for defining lesions and bitewings radiographs at baseline and follow ups.

## Exclusion criteria:

The exclusion criteria for present review were - Case reports, Case series, Cross-sectional studies, Invitro studies or Animal studies, Reviews & Abstracts, articles with incomplete data and patients with presence of any lesions were excluded, subjects with Enamel hypoplasia, one taking antibiotics, undergoing orthodontic treatment, having restoration/cavitation/chipping adjacent to white spot lesion, those who declined to participate and articles in any other language except English.

## Study selection and data extraction process:

Based on the defined inclusion and exclusion norms, two reviewers (BK, WN) independently reviewed the title and abstract of the selected papers. The reviewers read the papers separately and extracted the data using a data extraction form created specifically for this study. The following information was included on this form - author's name, year of publication, place, sample size, age of children, intervention, comparator, therapeutic effect, clinical and radiographic carious lesion progression and acceptability between the two reviewers were addressed by consulting a third reviewer (KS).

## Quality assessment of included studies:

This study's bias risk was assessed following the methods specified in the Cochrane Handbook for Systematic Reviews of Interventions [20]. Random sequence creation, allocation concealment, blinding tested in three groups: participant, operator and outcome assessor, inadequate outcome data, selective reporting and other biases were all considered.

## Results:

Figure 1 (see PDF) depicts the search results and study selection procedure. There were 41 preliminary records found in the electronic database searches: 33 from EBSCHOhost, 3 from The Cochrane Central Register of Controlled Trials and 4 from PubMed. When the references of the resulting papers were screened, no more acceptable research was located. The reference management programme was used to manually remove duplicates, resulting in 30 records. After reading the title and abstract, 17 were eliminated. After full text screening 09 articles excluded. Resultant 04 publications were eligible for qualitative synthesis. Table 1 (see PDF) outlines the technique followed by the included studies to evaluate and compare the efficacy of Resin infiltration with Fluoride varnish and Fluoride varnish alone. The chosen studies were carried out in the following three countries: Denmark (n = 2.5, 50%), Poland (01) and New Zealand (01).

## Characteristics of included studies (Table 1 - see PDF):

Research in dental materials and caries intervention continues to advance, with several notable contributions. Ekstrand *et al.* [21] demonstrated in a split-mouth randomized trial that combining resin infiltration with fluoride varnish significantly improved the management of proximal superficial carious lesions in primary molars compared to fluoride varnish alone. Similarly, Aziznezhad *et al.* [22] found *in vitro* that resin infiltrant, fluoride varnish, and nano-hydroxyapatite paste each had positive effects on surface hardness and bacterial adhesion of artificial enamel lesions, pointing to new preventive options. More recently, Almansouri *et al.* [23] evaluated different protective agents-including resin infiltration, fluoride, and biomimetic CPP-ACP-after orthodontic inter-proximal enamel reduction, confirming that newer biomimetic materials can support enamel resilience in such contexts. Collectively, these studies reflect a trend toward minimally invasive techniques and advanced materials for preserving enamel integrity and controlling early carious lesions.

## Quality assessment:

None of the papers included in this study were rated as high risk. At some of the factors that were evaluated, all four trials exhibited an unknown risk of bias. In this systematic review all studies had a low risk of bias (Table 2 - see PDF & Figure 2 - see PDF, 3 - see PDF).

## Discussion:

Dental caries remains one of the most widespread chronic diseases globally [25], with prevalence among infants and young children ranging from 3.3% to 61.1% across various populations [26, 27, 28, 29, 30, 31, 32- 33]. Multiple etiological factors contribute to its development, including feeding practices involving sugared beverages, prolonged breastfeeding and early-life dietary influences [27, 28, 29, 30, 31, 32, 33, 34, 35, 36- 37]. Additional determinants such as inadequate oral hygiene, increased *Streptococcus mutans* levels in dental plaque [38, 39], lower socioeconomic conditions-especially limited maternal education [32, 40] and geographical disparities [34] further exacerbate the disease burden. Recent global consensus meetings on caries detection and management have highlighted the limitations of current diagnostic approaches and emphasized a paradigm shift toward non-surgical management of early, non-cavitated lesions [41]. Accurate detection and diagnosis are essential to guide preventive and therapeutic decisions, wherein diagnosis refers to the recognition of disease based on signs and symptoms, while detection pertains to identifying those specific signs [42]. Among diagnostic modalities, bitewing radiography continues to serve as a key adjunct for identifying proximal carious lesions not visible during clinical examination. Radiographs are more sensitive than visual inspection for detecting proximal and occlusal caries, determining lesion depth and monitoring progression [43, 44]. However, for occlusal lesions, their diagnostic contribution is limited [45] and they are unable to reliably distinguish between active and arrested or between cavitated and non-cavitated lesions [46]. To improve consistency in visual assessments, the International Caries Detection and Assessment System (ICDAS) was developed in 2001 as a standardized framework integrating prior research findings into a unified clinical approach [47]. Drawing from foundational work and systematic reviews [48, 49, 50-51], ICDAS classifies carious lesions based on histological depth and visual appearance [52, 53], allowing for accurate early detection and longitudinal monitoring. Visual examination is performed with clean, dry teeth using a ball-ended explorer to enhance reproducibility [47, 54 and 55]. Therapeutic approaches for caries control range from preventive to micro-invasive strategies. The efficacy of topical fluoride applications is well established, with fluoride varnish use twice annually shown to significantly reduce caries incidence across age groups [56, 57, 58, 59, 60- 61]. Since its FDA approval in 1994 as a cavity liner and desensitizing agent [62], fluoride varnish has demonstrated a 40-56% reduction in caries occurrence [63] and some studies report up to 70-75% reduction with Duraphat formulations compared to other fluoride agents [64]. Its mechanism involves promoting remineralization of active lesions through the redeposition of calcium and phosphate ions as fluorapatite [65]. A more recent innovation, resin infiltration, represents a micro-invasive, non-surgical technique derived from early infiltration experiments with resorcinol-formaldehyde resins [66]. This approach utilizes low-viscosity resin to penetrate the porous enamel of non-cavitated lesions, sealing diffusion pathways and arresting lesion progression [67, 68]. Experimental studies have shown superior resin penetration in primary compared to permanent teeth following short application times [69]. Comparative clinical trials have consistently demonstrated the superior performance of resin infiltration when combined with fluoride varnish. Split-mouth randomized controlled trials have reported greater reductions in caries progression using combined resin infiltration and fluoride varnish compared to fluoride varnish alone [21]. Long-term follow-up studies have confirmed that infiltration is more effective than sealing or fluoride varnishing alone for managing early occlusal lesions [22]. Subsequent research employing dmfs and ICDAS II criteria has further validated the enhanced preventive efficacy of resin infiltration with fluoride varnish over varnish alone [23]. These findings have been corroborated by additional controlled trials demonstrating that resin infiltration significantly reduces caries progression in children, supporting its integration into preventive dentistry protocols [24]. Overall, the collective evidence underscores a progressive transition from purely preventive fluoride-based approaches to micro-invasive resin infiltration techniques, reflecting an evidence-driven evolution in modern caries management focused on early detection, non-surgical intervention and preservation of tooth structure [21- 69].

## Conclusion:

Resin infiltration combined with fluoride varnish demonstrates superior efficacy compared to fluoride varnish alone in reducing dental caries progression among school children. The synergistic action enhances enamel reinforcement and inhibits lesion advancement without adverse effects. This minimally invasive approach can be recommended as an effective preventive strategy in pediatric caries management.
